# Compound A, a Dissociated Glucocorticoid Receptor Modulator, Inhibits T-bet (Th1) and Induces GATA-3 (Th2) Activity in Immune Cells

**DOI:** 10.1371/journal.pone.0035155

**Published:** 2012-04-09

**Authors:** Ana C. Liberman, Maria Antunica-Noguerol, Viviane Ferraz-de-Paula, Joao Palermo-Neto, Carla N. Castro, Jimena Druker, Florian Holsboer, Marcelo J. Perone, Sarah Gerlo, Karolien De Bosscher, Guy Haegeman, Eduardo Arzt

**Affiliations:** 1 Laboratorio de Fisiología y Biología Molecular, Departamento de Fisiología y Biología Molecular y Celular, Facultad de Ciencias Exactas y Naturales, Universidad de Buenos Aires and Instituto de Investigación Biomedicina de Buenos Aires (IBioBA)-CONICET-Partner Institute of the Max Planck Society, Buenos Aires, Argentina; 2 Neuroimmunomodulation Research Group, FMVZ-USP, San Pablo, Brasil; 3 Max Planck Institute of Psychiatry, Munich, Germany; 4 Laboratory for Eukaryotic Gene Expression and Signal Transduction, Ghent University, Ghent, Belgium; 5 VIB Department of Medical Protein Research, UGent, Ghent, Belgium; Kaohsiung Chang Gung Memorial Hospital, Taiwan

## Abstract

**Background:**

Compound A (CpdA) is a dissociating non-steroidal glucocorticoid receptor (GR) ligand which has anti-inflammatory properties exerted by down-modulating proinflammatory gene expression. By favouring GR monomer formation, CpdA does not enhance glucocorticoid (GC) response element-driven gene expression, resulting in a reduced side effect profile as compared to GCs. Considering the importance of Th1/Th2 balance in the final outcome of immune and inflammatory responses, we analyzed how selective GR modulation differentially regulates the activity of T-bet and GATA-3, master drivers of Th1 and Th2 differentiation, respectively.

**Results:**

Using Western analysis and reporter gene assays, we show in murine T cells that, similar to GCs, CpdA inhibits T-bet activity via a transrepressive mechanism. Different from GCs, CpdA induces GATA-3 activity by p38 MAPK-induction of GATA-3 phosphorylation and nuclear translocation. CpdA effects are reversed by the GR antagonist RU38486, proving the involvement of GR in these actions. ELISA assays demonstrate that modulation of T-bet and GATA-3 impacts on cytokine production shown by a decrease in IFN-γ and an increase in IL-5 production, respectively.

**Conclusions:**

Taken together, through their effect favoring Th2 over Th1 responses, particular dissociated GR ligands, for which CpdA represents a paradigm, hold potential for the application in Th1-mediated immune disorders.

## Introduction

Glucocorticoids (GCs) are the most potent and frequently used anti-inflammatory drugs for a variety of Th1- and Th2-mediated immune disorders. Nevertheless, long-term applications are often complicated by severe adverse effects [1]. GCs act via binding to the glucocorticoid receptor (GR), a transcription factor (TF) belonging to the nuclear receptor superfamily. It is widely accepted that the desired anti-inflammatory effects of GCs are caused by the interaction of the monomeric GR with the activity of other TFs that drive proinflammatory gene expression, whereas the direct binding of GR to GC response elements (GREs) resulting in the direct transcription of target genes is mostly associated with well-known endocrine side effects [2]. This has led to the search for selective GR modulators, such as dissociated GR ligands, that selectively transrepress and which are predicted to reduce the appearance of a wide range of side effects. As the quest for dissociated steroidal GR ligands did not quite live up to expectations, there is currently a renewed interest of the pharmaceutical industry to find non-steroidal selective GR modulators with a reduced side effect profile yet maintaining their therapeutic efficacy [3].

Compound A (CpdA) is a stable analog of the hydroxy phenyl aziridine precursor found in the Namibian shrub *Salsola tuberculatiformis* Botschantzev [4]. CpdA is a clearly dissociating compound [4]. This means that it does not stimulate GRE-driven gene expression. It has been shown that CpdA and the synthetic GC dexamethasone (Dex) interact with the GR with comparable affinities, in the nanomolar range, but varying dependent on the cell type [4], [5]. The specific gene-repressive effect of CpdA depends on the presence of functional monomeric GR [6], displaying a differential phosphorylation status as compared to Dex [4]. The anti-inflammatory mechanism of CpdA involves both a reduction of DNA-binding activity, as well as an interference with the transactivation potential of NF-κB [4], which plays a central role in inflammation. Analysis of diverse mouse models of inflammatory and autoimmune diseases further supports the idea that CpdA has a potent anti-inflammatory activity and particularly lacks diabetogenic and bone metabolism side effects when applied *in vivo* compared with GCs [4], [6]–[10].

The adaptive immune response is triggered when T cells recognize antigens, which have been presented by antigen presenting cells. GATA-3 [11] is a master TF involved in Th2 development [12]. Th2 cytokines promote B cell-mediated humoral immunity against extracellular pathogens [13]. Th2 cytokines include IL-4, IL-5, IL-13 and IL-10. Ectopic expression of GATA-3 in developing and fully committed Th1 cells gives rise to Th2 cytokine production as well as Th1 cytokine inhibition [14]. GATA-3 regulates Th2 cytokine expression not only at the transcription level, by directly binding to the IL-5-promoter, but also by remodeling the chromatin structure and opening the IL-4 locus [15]. As a master control, GATA-3 stabilizes the Th2 phenotype in three ways [16]. First, GATA-3 shuts down Th1 development by down-regulation of STAT4/IL-12Rbeta2 chain or T-bet. Second, GATA-3 augments its own expression by a positive feedback autoregulation [17]. Third, GATA-3 favors selective growth of Th2 cells [16]. In Th2 cells [18], cAMP induces GATA-3 phosphorylation via p38 MAPK and stimulates GATA-3-dependent promoter activities [18], [19]. Intracellular increments of cAMP levels in Th cells are associated with an augmentation of Th2 cytokine production via GATA-3 and protein kinase A (PKA) activation [20].

T box expressed in T cells (T-bet) is a Th1 specific TF that controls the expression of the potent proinflammatory cytokine IFN-gamma (IFN-γ) [21], hallmark of Th1 cell-mediated immunity [22], [23]. Over-expression of T-bet into primary T cells or even fully polarized Th2 cells is able to generate IFN-γ-producing Th1 cells, concomitant with an inhibition of the production of the Th2 cytokines IL-4 and IL-5 [21]. T-bet deficient mice show normal lymphoid development, but profound defects in mounting a Th1 immune response and a corresponding increase in Th2 cytokines [24]. T-bet may down-regulate GATA-3 function either by regulating its expression or by inhibiting its activity [21], [25].

We have previously described that GCs inhibit the transcriptional activity of T-bet [26] by a transrepression mechanism involving a protein-protein interaction between the activated GR and T-bet, resulting in a diminished DNA binding [26]. Also the master Th2 TF GATA-3 is inhibited by GCs, yet via a different molecular mechanism. GCs inhibit GATA-3 activity by the inhibition of p38 MAPK-induced GATA-3 phosphorylation and its nuclear translocation [27], [28]. Therefore, by suppressing Th1 responses to a stronger extent than Th2, GCs favor a shift from a Th1 towards a Th2 profile, which might have relevant implications in the treatment of Th1-polarized immune disorders [26], [29].

In order to discover novel targets in relevant immune-modulatory pathways that may be differentially affected by nonconventional GR modulators such as CpdA, and hence may become of direct clinical relevance, a detailed understanding of the molecular mechanism underlying the immune-modulatory effects of GCs and dissociated GCs in immune cells is a prerequisite. Although recent data regarding the effect of CpdA on the expression of Th cytokines in various animal models of immune diseases has been presented [7], [8], the molecular mechanism underlying differential effects of a selective modulation of GR in immune cells on downstream TF targets has not yet been clarified. To this purpose, we investigated how CpdA- and Dexamethasone (Dex)-activated GR differentially affect the activity of key TFs involved in the regulation and final outcome of Th-mediated immune responses.

## Results

### CpdA Inhibits the Transcriptional Activity of T-bet

**Figure 1:**
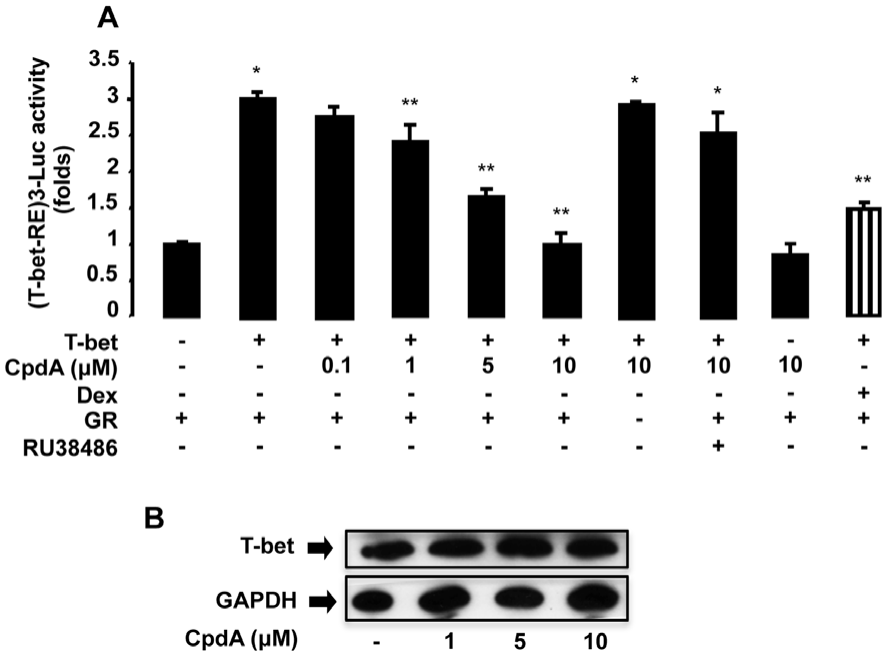
Compound A inhibits T-bet transcriptional activity. *A,* EL4 cells were transfected with 9 µg of a reporter plasmid which contains T-bet response elements upstream of the luciferase gene (T-bet-RE-Luc) with or without 9 µg of T-bet and GR expression vectors. After 16 h in culture, cells were stimulated for 5 h with or without Compound A (CpdA, 0.1, 1, 5 and 10 µM), Dexamethasone (Dex, 10 nM) or the GR specific antagonist RU38486 (1 µM). Results, as folds, normalized to β-galactosidase activity, are expressed as mean ± SEM (n  =  6, * p<0.001 vs. basal without T-bet, ** p<0.05 vs. T-bet without CpdA), averaged from three independent experiments. *B,* Lysates obtained from transfection experiments performed under similar conditions as mentioned above, were prepared for T-bet analysis by Western Blot. Single bands corresponding to T-bet were obtained. GAPDH signal was used as loading control; one of three independent experiments with similar results is shown.

We established before that conventional GR activation, through the use of the classic GC Dexamethasone (Dex), can negatively impact on the activity of T-bet [26]. At present it is unknown whether or not selective GR modulation, through the use of the dissociated GR modulator CpdA, can regulate the activity of immune-regulatory TFs, other than NF-κB. Hence, we investigated whether CpdA could also negatively regulate the transcriptional activity of the key Th1 TF T-bet. Transfection of EL4 T cells with T-bet response elements cloned upstream of the luciferase gene (T-bet-RE-Luc) together with GR (CMV-hGR) and T-bet (pcDNA3-T-bet) expression vectors in the presence of increasing amounts of CpdA led to a dose-dependent inhibition of T-bet’s transcriptional activity (Fig. 1A), similar to the effect we have previously reported for GCs (Fig. 1A, striped bar) [26]. The antagonistic effect exerted by RU38486 proved that the ligand-binding domain of GR is involved in CpdA-mediated inhibition of T-bet activity (Fig. 1A). In addition, no effect of CpdA was observed when the GR was absent from the transfection experiments. Similar results were obtained using the human Jurkat T cell line (data not shown).

Western Blot assays performed under similar conditions as mentioned above, showed that CpdA does not affect the over-expressed protein levels of T-bet (Fig. 1B), strongly suggesting that CpdA directly targets T-bet at the transcriptional level.

### CpdA Inhibits the Transcriptional Activity of T-bet via a Transrepression Mechanism

**Figure 2:**
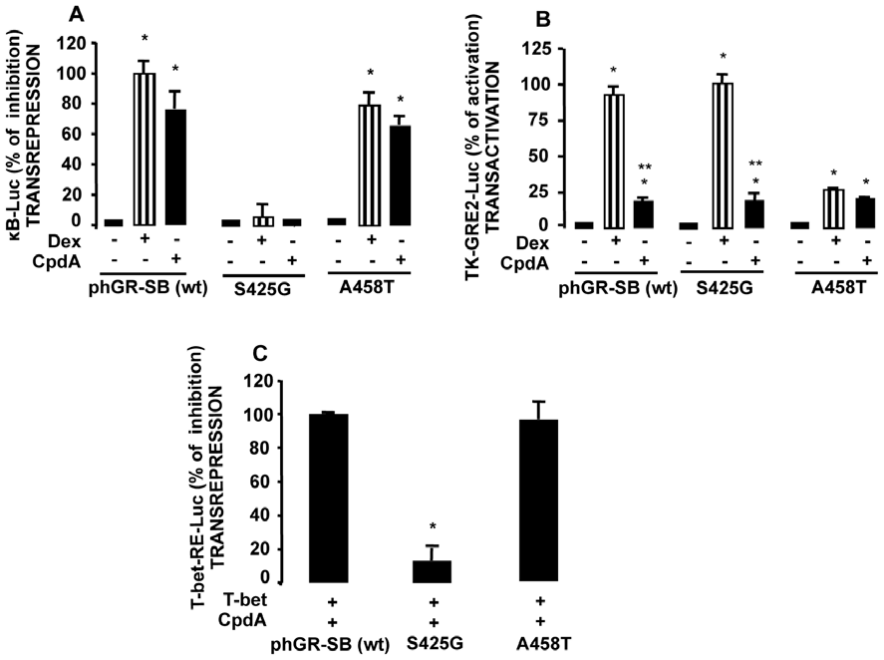
Compound A inhibits T-bet transcriptional activity by transrepression. *A,* EL4 cells were cotransfected with 9 µg of κB-Luc reporter plasmid and 9 µg of Rel A plus 9 µg of phGR-SB (wt) wild-type GR expression vectors or the GR mutants A458T or S425G. After 16 h, cells were stimulated for 8 h with IL-1β (10 ng/ml), which induces κB-Luc activity, and with CpdA (10 µM) or Dex (100 nM). Results, as % of inhibition of κB-Luc, normalized to β-galactosidase activity, are expressed as mean ± SEM (n  =  3, * p<0.001 vs. Rel A plus IL-1β without Dex or CpdA), of one representative experiment of three independent experiments with similar results. *B*, EL4 cells were transfected with 9 µg of a reporter plasmid containing two palindromic GR-binding sites coupled to the TK promoter reporter plasmid (TK-GRE2-Luc) plus 9 µg of phGR-SB (wt) wild-type GR expression vector or the GR mutants A458T or S425G. After 16 h, cells were stimulated for 5 h with CpdA or Dex. Results, as % of activation of TK-GRE2-Luc, normalized to β-galactosidase activity, are expressed as mean ± SEM (n  =  3, * p<0.05 vs. basal without Dex or CpdA, ** p<0.05 vs. Dex treated wild-type or S425G mutant GR), of one representative experiment of three independent experiments with similar results. *C,* EL4 cells were transfected with 9 µg of T-bet-RE-Luc reporter plasmid and with 9 µg of T-bet and wild-type GR expression vectors (ph-GR-SB) or the GR mutants A458T or S425G. After 16 h, cells were stimulated for 5 h with CpdA. Results, as % of inhibition of T-bet-RE-Luc, normalized to β-galactosidase activity, are expressed as mean ± SEM (n  =  3, * p<0.001 vs. ph-GR-SB or A458T-mediated T-bet-RE-Luc relative inhibition) of one representative experiment of three independent experiments with similar results.

Taking into account that GCs inhibit T-bet activity via transrepression and that CpdA does not transactivate GRE-dependent genes but can transrepress cytokine genes [4], we analyzed whether the underpinning mechanism by which CpdA inhibits T-bet activity could be transrepression. To adress this hypothesis we used two extensively described GR mutants [26], [30], [31]. A458T is a D-loop dimerization interface mutation that blocks GR dimerization and activation of transcription [31]. S425G is a DBD mutant that has a serine to glycine substitution at position 425, which removes a hydroxyl group supposed to alter bondings between the zinc finger domain and other proteins and hence disrupts transrepression [30]. Transfection and recombinant reporter gene analyses in EL4 cells show that CpdA-activated wild-type GR, but not CpdA-activated S425G GR, the GR mutant defective for transrepression, was able to transrepress κB-Luc activity (Fig. 2A). On the contrary, CpdA retained its transrepressive capacity when triggering the A458T GR-transactivation defective mutant (Fig. 2A). Neither the wild-type nor the mutant GRs could transactivate TK-GRE2-Luc in the presence of CpdA (Fig. 2B), confirming its dissociated activity also in immune cells. As expected, Dex, the classic GC, behaved similar as CpdA in the transrepression assays (Fig. 2A, striped bars), and opposite to CpdA in the transactivation assays (Fig. 2B, striped bars).

Concerning the effect of a dissociative GR modulation on the T-bet-RE-Luc reporter gene activity, we found that both CpdA-activated wild-type GR and A458T, the GR-transactivation mutant, can strongly inhibit T-bet activity. On the contrary, CpdA-activated S425G GR mutant displayed a reduced ability in repressing T-bet activity, when compared to the strong inhibition exerted by the CpdA-treated wild-type and A458T mutant. These data, using mutant GR and/or the dissociative activity of a GR ligand, are in line with a mechanism whereby the transrepressive function of GR can be exclusively held responsible for the GR-mediated inhibition of T-bet activity.

### CpdA Inhibits T-bet-driven IFN-γ Gene Expression and Protein Production

**Figure 3:**
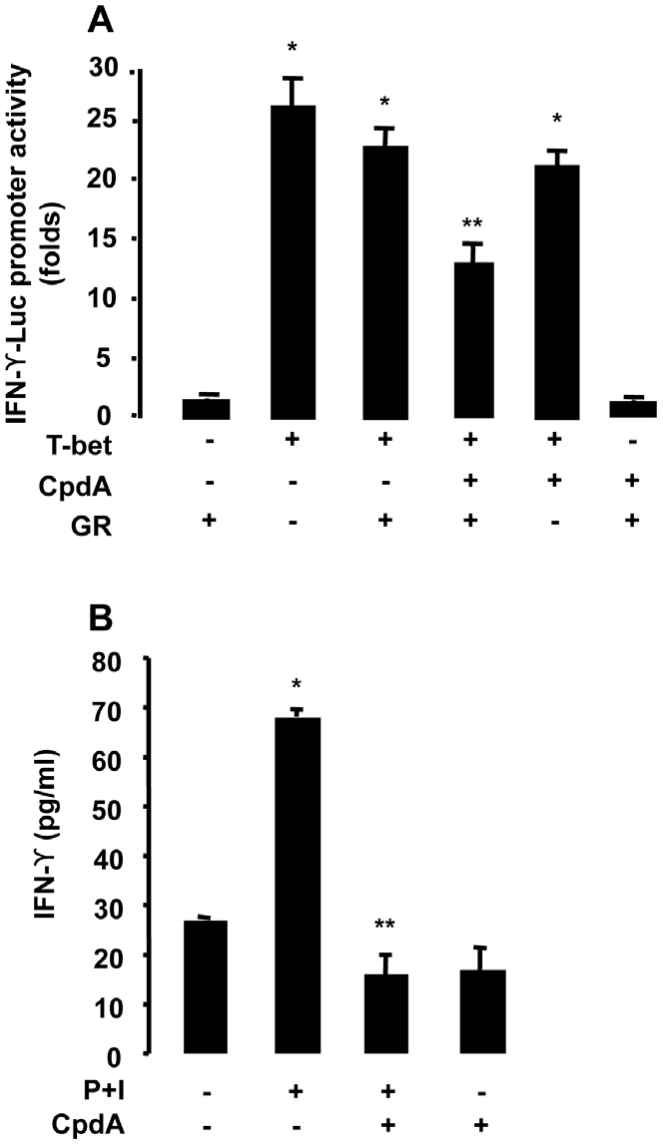
Compound A inhibits IFN-γ promoter activity and cytokine production. *A,* EL4 cells were cotransfected with 9 µg of IFN-γ promoter-driven luciferase plasmid (IFN-γ-Luc) plus 9 µg of GR expression vector and with 9 µg of T-bet expression vector. After 16 h in culture, cells were stimulated for 5 h with Compound A (CpdA, 10 µM). Results, as folds, normalized to β-galactosidase activity, are expressed as mean ± SEM (n  =  6, * p<0.001 vs. basal without T-bet, ** p<0.001 vs. T-bet without CpdA), averaged from three independent experiments. *B,* Purification of T cells was achieved by a conventional technique involving cell adhesion to plastic and then to a nylon wool column and alternatevely by FACS sorting. Purified T cells were activated with PMA (P) and Ionomycin (I) during 24 h and then incubated with CpdA for 5 h. Supernatants were used to measure mouse IFN-γ according to the manufacturer’s instructions by ELISA. Results are expressed as mean ± SEM (n  =  4, * p<0.001 vs. basal without CpdA, ** p<0.001 vs. P+I without CpdA) of one representative experiment of three independent experiments with similar results.

We tested the functional relevance of CpdA-mediated inhibition of T-bet transcriptional activity on IFN-γ, hallmark of Th1-mediated immune responses [21]. To investigate whether CpdA-dependent inhibition of T-bet activity can be observed in a functionally relevant promoter context, i.e. on the IFN-γ promoter, EL4 cells were transfected with a -3447-IFN-γ promoter cloned upstream of the luciferase gene together with the GR expression vector. Over-expression of T-bet resulted in a strong increase of the luciferase activity, whilst cotransfection of GR in the presence of CpdA led to the inhibition of IFN-γ-promoter activity (Fig. 3A). No effect of CpdA was observed when GR was absent from the transfection experiments. Under the same conditions as in the transfection experiments, Western Blots were carried out to analyze T-bet protein expresion. No changes in the over-expressed T-bet protein levels were observed following CpdA treatment (data not shown), suggesting that the CpdA-mediated inhibition of T-bet is at the transcriptional level and a relevant mechanism in the regulation of the IFN-γ promoter. Next, we analyzed whether the transcriptional repression of CpdA could also be reflected in a diminished IFN-γ protein production in purified CD4+ T cell cultures. Indeed, Fig. 3B shows that CpdA strongly inhibits IFN-γ production. Similar results were obtained in non-adherent and in total splenocyte cultures (data not shown).

### CpdA Induces the Transcriptional Activity of GATA-3 by Signaling Through p38 MAPK

**Figure 4:**
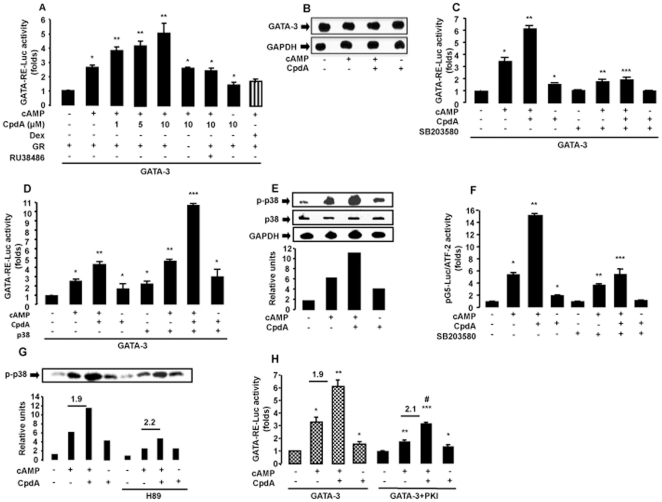
Compound A induces GATA-3 transcriptional activity by signaling through p38 MAPK. *A,* EL4 cells were transfected with 9 µg of a reporter plasmid, which contains GATA-3 response elements upstream of the luciferase gene (GATA-RE-Luc) and with 9 µg of GATA-3 and GR expression vectors. After 16 h, cells were stimulated for 5 h with Compound A (CpdA, 1, 5 and 10 µM), Dexamethasone (Dex, 10 nM), cAMP (0.3 mM) and with the GR specific antagonist RU38486 (1 µM). n  =  6, * p<0.001 vs. GATA-3 without CpdA and cAMP, ** p<0.001 vs. GATA-3 with cAMP and without CpdA. *B,* Lysates obtained from transfection experiments performed under similar conditions as mentioned above, were analysed by Western Blot. GAPDH was used as loading control. *C,* EL4 cells were transfected with 9 µg of GATA-RE-Luc reporter and with 9 µg of GATA-3 and GR expression vectors. After 16 h, cells were stimulated for 5 h with CpdA (10 µM) and cAMP. Also, EL4 cells were pretreated during 1 h with the p38 MAPK inhibitor, SB203580 (10 µM). n  =  6, * p<0.001 vs. GATA-3 without cAMP and CpdA, ** p<0.001 vs. GATA-3 with cAMP and without CpdA, *** p<0.001 vs. GATA-3 with cAMP and CpdA. *D,* EL4 cells were transfected with 9 µg of GATA-RE-Luc reporter and 9 µg of GATA-3, GR and p38 MAPK expression vectors. After 16 h, cells were stimulated for 5 h with CpdA and cAMP. n  =  6, * p<0.001 vs. GATA-3 without cAMP and CpdA, ** p<0.001 vs. GATA-3 with cAMP and without CpdA, *** p<0.001 vs. GATA-3 with cAMP and p38. *E,* EL4 cells were transfected with 20 µg of the GR expression vector. After 16 h, EL4 cells were pretreated during 30 minutes with CpdA and then with cAMP during 25 minutes. Cell lysates were prepared for Western Blot analysis against phospho-p38 (p-p38) MAPK. GAPDH and total p38 signals were used as loading controls. Lower panel: NIH Image semiquantification. *F,* EL4 cells were transfected with 9 µg of pFA-ATF2 and 9 µg of pG5-Luc reporter plasmid, and the GR expression vector. After 16 h, cells were stimulated for 5 h with CpdA and cAMP. Also, EL4 cells were pretreated during 1 h with the p38 MAPK inhibitor, SB203580. n  =  6, * p<0.001 vs. basal without cAMP and CpdA, ** p<0.001 vs. cAMP without CpdA, *** p<0.001 vs. cAMP and CpdA. For all the transfections experiments, results, as folds, normalized to β-galactosidase activity, are expressed as mean ± SEM, averaged from three independent experiments.

To determine whether CpdA also regulates the transcriptional activity of the master Th2 TF GATA-3, we transfected EL4 cells with a GATA-3-dependent reporter gene construct (GATA-3-RE-Luc) together with GR (CMV-hGR) and GATA-3 (pcDNA3-GATA-3) expression vectors (Fig. 4A). Contrary to the effect we have previously described for GCs [27], increasing amounts of CpdA, in the presence of cAMP, led to a dose-dependent induction of GATA-3 activity (Fig. 4A).

The reversion by the specific antagonist RU38486 proved that the ligand-binding module of the GR is involved in the CpdA-mediated induction of GATA-3 activity (Fig. 4A). In addition, no effect of CpdA was observed when GR was not present in the transfection experiment. Similar results were obtained using Jurkat cells (data not shown).

As the induction of GATA-3 activity could be the consequence of an increased GATA-3 protein expression, we analyzed over-expressed GATA-3 protein levels by Western Blot under the same conditions as in the transfection experiments. We observed no changes in GATA-3 protein levels, strongly suggesting that CpdA induces GATA-3 transcriptional activity (Fig. 4B).

As previously described, cAMP treatment enhances the activity of GATA-3 via p38 MAPK induction [18]–[20], [27]. Therefore, in order to study the effect of CpdA on the activity of this kinase, EL4 cells were transfected with a GATA-3-dependent reporter gene construct (GATA-3-RE-Luc) together with GR (CMV-hGR) and GATA-3 (pcDNA3-GATA-3) expression vectors. CpdA-induced GATA-3 activity was inhibited in the presence of the kinase inhibitor SB203580 (Fig. 4C), supporting the involvement of the p38 MAPK pathway for CpdA-induced GATA-3 activity. Overexpressing p38 MAPK, using a p38 expression vector (pcEFL-p38α), resulted in a further increase of CpdA- and cAMP-induced GATA-3 activities. These results confirm an important role for p38 MAPK in driving the activity of GATA-3, elicited by two different (Fig. 4D).

To test whether the molecular mechanism by which CpdA induces the transactivation of GATA-3 involves CpdA-mediated p38 phosphorylation, we performed Western Blot assays in EL4 cells transfected with a GR expression vector (CMV-hGR) in the presence of CpdA and cAMP (Fig. 4E). The results demonstrate that CpdA already on its own strongly induces p38 phosphorylation, and that the effect is enhanced in the presence of cAMP.

Phosphorylation and activation of the downstream TF ATF2 is known to reflect an activation of the p38 MAPK signaling pathway [32]. A chimeric trans-activator protein containing ATF2 fused to the DNA binding domain of the yeast transcriptional activator GAL4 (pFA-ATF2) was transiently transfected into cells with a luciferase reporter containing five copies of a GAL4 DNA binding element upstream of a TATA box and the luciferase gene (pG5-Luc). Thus, by monitoring the activity of the pG5-Luc reporter, the activation of ATF2 by p38 MAPK was followed. Fig. 4F shows that CpdA strongly induces the reporter activity and SB203580 inhibits this induction further confirming that CpdA is able to induce p38 MAPK activity.

PKA, MEK-1 and JNK pathways are not involved as shown by the lack of inhibition of CpdA-induced GATA-3 activity using the specific inhibitors of these kinases H89, PD98059 and SP600125 respectively (data not shown).

### CpdA Induces Phosphorylation and Nuclear Translocation of GATA-3

**Figure 5:**
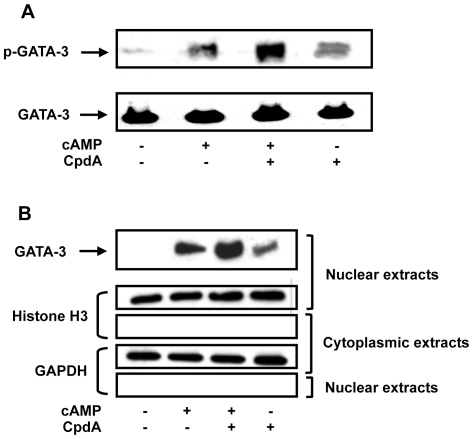
Compound A induces GATA-3 phosphorylation and nuclear translocation. *A,* EL4 cells were transfected with 15 µg of the GR and 15 µg of GATA-3 expression vectors. After 16 h in culture, EL4 cells were pretreated during 30 minutes with or without Compound A (CpdA, 10 µM) and then treated under basal or activated conditions using cAMP (0.3 mM) during 25 minutes. Cell lysates were prepared for Western Blot analysis against phospho-GATA-3 (p-GATA-3) (bands of 55 kDa). Total GATA-3 signal was used as loading control. One out of three independent experiments with similar results are shown. *B,* EL4 cells were transfected with 15 µg of the GR and 15 µg of GATA-3 expression vectors. After 16 h in culture, EL4 cells were pretreated during 30 minutes with or without CpdA (10 µM) and then treated under basal or activated conditions using cAMP (0.3 mM) during 25 minutes. Cell nuclear and cytoplamic extracts were prepared for GATA-3 analysis by Western Blot. Histone H3 and GAPDH signals were used as nuclear and cytoplasmic extracts control respectively. One out of three independent experiments with similar results are shown.

It has been reported that p38 MAPK induces GATA-3 phosphorylation and nuclear translocation, which impacts on the transcriptional activity of GATA-3 [19]. To directly investigate whether the effect of CpdA on p38-mediated GATA-3 activity involves the induction of GATA-3 phosphorylation [27], [28], Western Blots were performed using EL4 cells stimulated under basal, cAMP- and CpdA-activating conditions using a specific antibody against phospho-GATA-3 (p-GATA-3). As shown in Fig. 5A, although CpdA does induce a slight GATA-3 phosphorylation on its own; in the presence of cAMP, this effect is significantly enhanced. As expected, also cAMP on its own induces phosphorylation of GATA-3. Whole extract lysates were used as controls for total GATA-3 expression (Fig. 5A).

To address whether CpdA further enhances the nuclear translocation of GATA-3, we performed Western Blot experiments using cytoplasmic and nuclear extracts. Fig. 5B shows that as previously reported, cAMP induces GATA-3 nuclear translocation. An additional treatment with CpdA further induces this translocation. A specific antibody against Histone H3 was used as control to demonstrate the purity of the nuclear extracts and GAPDH was used as control of the cytoplasmic extracts (Fig. 5B).

### CpdA Induces GATA-3-driven IL-5 Gene Expression and Protein Production

**Figure 6:**
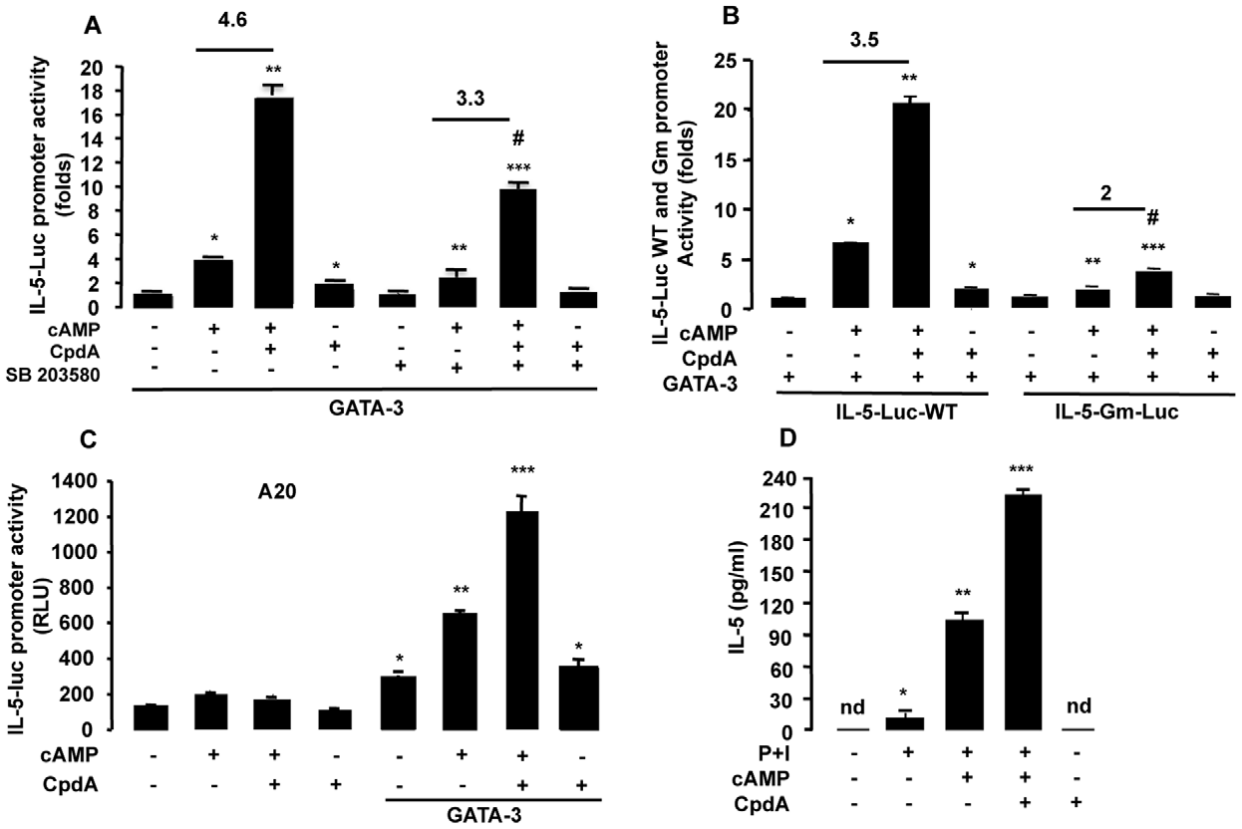
Compound A inhibits IL-5 promoter activity and cytokine production. *A,* EL4 cells were cotransfected with 9 µg of IL-5-Luc reporter plasmid plus 9 µg of GR and GATA-3 expression vectors. After 16 h, cells were stimulated for 5 h with CpdA (10 µM) and cAMP. Also, EL4 cells were pretreated during 1 h with p38 inhibitor, SB203580 (10 µM). n  =  6, * p<0.001 vs. GATA-3 without cAMP and CpdA, ** p<0.001 vs. GATA-3 with cAMP and without CpdA, *** p<0.001 vs. GATA-3 with cAMP and CpdA, # p<0.001 vs. GATA-3 with cAMP and SB203580 and without CpdA. *B,* EL4 cells were cotransfected with 9 µg of the wild-type or GATA-3 mutated-binding site on the IL-5 promoter (IL-5-Luc-WT and IL-5-Gm-Luc) plus 9 µg of GR and GATA-3 expression vectors. After 16 h, cells were stimulated for 5 h with CpdA and cAMP. n  =  6, * p<0.001 vs. GATA-3 without cAMP and CpdA on IL-5-Luc WT, ** p<0.001 vs. GATA-3 with cAMP and without CpdA on IL-5-Luc WT, *** p<0.001 vs. GATA-3 with cAMP and CpdA on IL-5-Luc WT, # p<0.05 vs. GATA-3 with cAMP and without CpdA on IL-5-Gm-Luc. *C,* A20 cells were cotransfected with 9 µg of IL-5-Luc reporter plus 9 µg of GR and GATA-3 expression vectors. After 16 h, cells were stimulated for 5 h with CpdA and cAMP. n  =  6, * p<0.001 vs. basal, ** p<0.001 vs. GATA-3, *** p<0.001 vs GATA-3 with cAMP. *D,* Purification of T cells was achieved as described in Materials and Methods. Cells were activated with PMA (P), Ionomycin (I) and cAMP during 24 h and then with CpdA for 5 h. Supernatants were used to measure mouse IL-5 by ELISA. n  =  4, * p<0.05 vs. basal without P+I, ** p<0.001 vs. P+I without cAMP, *** p<0.001 vs. P+I and cAMP, of one representative experiment of three independent experiments with similar results. For the transfections experiments, results, as folds, normalized to β-galactosidase activity, are expressed as mean ± SEM, averaged from three independent experiments.

To investigate the possible involvement of CpdA-dependent induction of GATA-3 activity in the regulation of IL-5, a central Th2 cytokine, EL4 cells were transfected with the IL-5 promoter cloned upstream of the luciferase gene together with GR (CMV-hGR) and GATA-3 (pcDNA3-GATA-3) expression vectors. Over-expression of GATA-3 in the presence of CpdA led to strong induction of IL-5 activity (Fig. 6A). The involvement of p38 MAPK was evident because CpdA-induced GATA-3 activity was inhibited in the presence of the specific kinase inhibitor SB203580.

To confirm the involvement of GATA-3 on CpdA-mediated IL-5 promoter activity, we transfected EL4 cells with the proximal IL-5 promoter (−120 to +44) bearing its unique GATA-3 binding site (around position −72), or its mutated version on the GATA-3 binding site (IL5-Gm-luc) (Fig. 6B). CpdA induces the wild-type IL-5 promoter activity but its effect is strongly inhibited upon using the promoter variant containing the mutated GATA-3 binding site, confirming the importance of GATA-3 in mediating the CpdA-activated promoter activity.

The A20 mouse B lymphoma cell line, which does not express endogenous GATA-3 but expresses endogenous GR, was transfected with the IL-5 promoter-driven reporter gene construct in the presence or absence of a GATA-3 expression vector. There was no IL-5 promoter activity in the absence of GATA-3 and when GATA-3 is overexpressed, CpdA is able to further induce the activity of cAMP-stimulated GATA-3, confirming that the IL-5 promoter is induced by a CpdA-modulated GATA-3 activity (Fig. 6C).

Next, we analyzed whether the transcriptional activation of CpdA could also be reflected in an increase of IL-5 protein production in purified CD4+ T cell cultures. Indeed, Fig. 6D shows that CpdA strongly induces IL-5 production. Similar results were obtained in non-adherent and in total splenocyte cultures (data not shown).

## Discussion

In this work we describe the mechanisms of action following selective GR modulation in immune T cells. Hereto, we studied the effect of CpdA, as a paradigm for a dissociative GR activity, on the activity of key Th TFs that control the development and final outcome of the adaptive immune responses: T-bet, involved in Th1 cellular immunity; and GATA-3, involved in Th2 humoral immune responses.

Our findings demonstrate that in T cell lines, CpdA inhibits the transcriptional activity of T-bet via a mechanism involving GR-mediated transrepression, similar to the effect previously described for GCs [26]. On the other hand, different from the mechanism that was previously described for GCs on GATA-3 activity [27], CpdA induces the transcriptional activity of GATA-3 via an enhanced induction of p38 MAPK-mediated GATA-3 phosphorylation and subsequent nuclear translocation. Considering that in rats with experimental autoimmune neuritis, CpdA treatment increased the numbers of anti-inflammatory M2 macrophages and inhibited the mRNA expression of inflammatory cytokines [8], CpdA may have a dual effect on immune cells: 1) on macrophages to dampen an uncontrolled first line of defense, and 2) modulation of the Th1-Th2 balance in the second stage.

Since nothing is known yet about the regulation of key Th immune-regulatory TFs, we first investigated the role of CpdA on the transcriptional activity of T-bet in T cells. We transfected EL4 cells with a construct bearing T-bet response elements cloned upstream of the luciferase gene and found that CpdA strongly inhibits the transcriptional activity of T-bet. The actions of CpdA were reverted by the synthetic antagonist RU38486 which acts as a competitor for ligand binding to GR, unambiguously demonstrating direct GR involvement.

It is generally accepted that DNA binding and subsequent activation of gene expression requires the dimerization of GR and binding to a palindromic GRE [33]. Mutagenesis studies of the GR DBD showed that disruption of the D-loop abolishes the ability of GR to dimerize, thus inhibiting GC-mediated activation [34]. On the other hand, a GR mutant harboring a point mutation in the second zinc finger of the DBD was unable to repress stimulated NF-κB but still could activate target genes through GREs suggesting that activation and transrepression are separate phenomena. As mentioned before, S425G DBD mutant has a serine to glycine substitution at position 425, which removes a hydroxyl group supposed to alter bondings between the zinc finger domain and other proteins such as T-bet [26]. Our finding that the first zinc finger of the DBD is important for CpdA and GC-mediated inhibition of T-bet activity strongly suggests that this region is essential for transrepression between the GR and T-bet.

IFN-γ is a strong activator of inflammatory responses and cellular immunity. Studies of the IFN-γ gene promoter have shown that T-bet elements play an important role in the induction of transcription and production of this cytokine [21], [24], [35], [36]. It has been previously reported that CpdA inhibits IFN-γ mRNA expression and cytokine production in spinal cord and total splenocytes from mice with EAE [9] and also the mRNA expression in lymph nodes of rats with EAN [8]. Our data add onto these observations by demonstrating the inhibitory effect of CpdA on PMA and Ionomycin-stimulated IFN-γ production in purified CD4+ T cells. Moreover, we continue to show that the underlying molecular mechanism of IFN-γ gene inhibition following selective GR modulation involves the inhibition of T-bet activity. Considering the previously reported inhibition of NF-κB by CpdA [4], we speculate that other TFs besides T-bet, such as NF-κB, may also be involved and may contribute to the inhibition of IFN-γ cytokine production by CpdA. If we consider that IFN-γ is a major activator of macrophages and that CpdA inhibits IFN-γ production by T cells, the inhibitory effect of CpdA on macrophage-mediated inflammatory responses may be reinforced by the inhibition of IFN-γ production and therefore may further favor the resolution of inflammation.

Upon investigating the effect and functional consequences of CpdA on the activity of the Th2 key TF GATA-3 we found that CpdA induces GATA-3 activity on its own GATA-3 response elements. GR specificity was guaranteed by showing that the CpdA effect could be reversed by the synthetic antagonist RU38486 that acts as a competitor for binding to GR.

GCs inhibit GATA-3 activity by inhibition of p38 MAPK-mediated GATA-3 phosphorylation and hindering its nuclear translocation [27], [28]. Bearing this in mind, we studied the effect of CpdA on p38 MAPK signaling and its subsequent effect on GATA-3 activity. Using the p38 MAPK inhibitor SB203580, we showed that CpdA induces GATA-3 transcriptional activity by induction of p38 MAPK phosphorylation. Intracellular increments of cAMP levels are associated with an augmentation of Th2 cytokine production via GATA-3 and PKA activation [20]. Therefore we performed transfection and Western Blot experiments using the heat stable inhibitor of the PKA (PKI) or the PKA inhibitor H89 and demonstrated that CpdA induces GATA-3 activity independently of PKA (data not shown). CpdA induction of p38 MAPK phosphorylation is mediated by the GR because pre-incubation with RU38486 reversed this effect (data not shown). In addition, Western Blot experiments using a specific antibody against phosphorylated GATA-3 show that CpdA strongly induces GATA-3 phosphorylation.

GCs induce the expression of mitogen-activated protein kinase (MAPK) phosphatase-1 (MKP-1), the endogenous inhibitor of p38 MAPK, which is necessary for GATA-3 nuclear translocation [27], [28]. To study whether the induction of p38 MAPK by CpdA could be due to the inhibition of MKP-1 expression, we checked the levels of endogenous MKP-1. These experiments however showed that MKP-1 protein levels are not affected by CpdA (data not shown). This is in accordance with previous results showing that MKP-1 expression on primary microglia and astrocytes is not affected by the addition of CpdA [9]. We also tested the possibility that the mitogen- and stress-activated protein kinase- 1 (MSK1), which has been shown to be activated by p38 MAPK and inhibited by GC [37], was implicated in CpdA induction of GATA-3 phosphorylation. However, no MSK1 induction was found in CpdA-stimulated EL4 cells (data not shown).

Because GATA-3 directly controls the expression of IL-5 gene, by binding to elements on the –70 to –59 region [38] on its minimal promoter, we used as a readout of CpdA effect on GATA-3 activity the IL-5 promoter. We describe for the first time that selective modulation of GR, by means of the transrepression-favouring GR modulator CpdA, strongly induces IL-5 gene and cytokine production via an enhancement of the activation of GATA-3. We show that CpdA induces GATA-3 activity, driving the IL-5 promoter. The addition of SB203580 inhibits this induction, again suggesting an involvement of the p38 MAPK pathway. GATA-3 binding site mutations in the IL-5 promoter further confirm that GATA-3 is a likely target of CpdA, and is needed in order to enhance the activity of this promoter. The mechanism described here does not rule out that in a full physiological promoter context additional indirect effects may occur. For example, a combination of CpdA-mediated induction of other kinase pathways implicated in GATA-3 phosphorylation or in p38 MAPK induction such as the upstream MAPK kinases MKK3 and MKK6 [39], an interaction with other TFs involved in the transcriptional complex of GATA-3, a recruitment and activation of other TFs to the IL-5 promoter [40] or even a recruitment of co-activators by GR may all contribute to the overall induction of IL-5 gene.

In lymph nodes of rats with EAN, CpdA inhibits mRNA expression of the IFN-γ gene and at the same time induces Th2-type cytokines expression [8]. In line with this observation, our data showing that CpdA inhibits T-bet activity which impacts on IFN-γ gene, and at the same time induces GATA-3 mediated Th2-type cytokine production, may contribute to provide a molecular understanding for the previously described immunosuppressive action of CpdA in EAN.

Summing up, CpdA directly inhibits T-bet activity and IFN-γ production. On the other hand, considering the mutual inhibitory action between T-bet and GATA-3 [14], [17], [25], [41], the inhibition of the Th1 profile by CpdA may be reinforced by inducing GATA-3 and Th2-type cytokine production, which in turn may further inhibit Th1 development. T-bet is known for its important role in inflammation and autoimmune disorders such as Inflammatory Bowel Disease, Multiple Sclerosis, Inflammatory Arthritis and Diabetes [42]. Therefore, CpdA-mediated inhibition of the Th1 phenotype may be helpful to understand the anti-inflammatory role reported for CpdA in many of these diseases and holds potential for the application of novel drugs which retain CpdA-like characteristics in other inflammatory and Th1-mediated autoimmune disorders. Indeed, although CpdA is not druggable as such, its molecular regulation of GR activities presents important insights on GR biology in immune regulation, and confidence that the dissociative ligand hypothesis remains of great value. However a clinical application of CpdA-like molecules for the treatment of inflammatory and Th1-mediated autoimmune diseases must be carefully studied taking into account the association of GATA-3 and IL-5 with allergic diseases such as asthma pathogenesis. Therefore, a balance considering the reduction of T-bet-mediated autoimmune and inflammatory responses together with the induction of Th2 responses should be considered while setting up the clinical use of GR-ligands that harbor similar characteristics as CpdA.

## Materials and Methods

### Splenocytes and Cell Line Cultures

Studies employing animals were conducted according to the NIH guidelines and were approved by the Animal Research and Care Committee (CICUAL # 2009/044) at the School of Exact and Natural Sciences, University of Buenos Aires.

Spleens were removed aseptically from naive BALBc mice and dispensed through a metal mesh in order to obtain single-cell suspensions. Splenocytes were resuspended at a density of 2.5 × 10^6^ cells and plated in 6-well plates. Purification of T cells was achieved as described [26], [27] using a nylon wool column. Monocyte contamination was verified to be less than 1%. Purity of the cell population was assayed by immunofluorescence using specific monoclonal antibodies (Serotech Laboratories Limited, Toronto, Canada) [26]: CD2, CD4, CD8, CD14, CD19, and CD45, which define antigens on T cells/NK cells, Th lymphocytes, T cytotoxic/suppressor cells, monocytes/macrophages, B cells and leukocytes (leukocyte common antigen), respectively. Where indicated, these purified murine T cells were further purified by FACS (FACSAriaII, Becton Dickinson, San Jose CA) using anti-CD4 antibody (BD Pharmingen, San Jose, CA) to 95–99% of purity. To determine cytokine secretion cells were seeded and stimulated at the beginning of the culture with 10 ng/ml PMA, 500 ng/ml ionomycin (I) for 24 h and then incubated with 10 µM CpdA for 6, 12 or 24 h. Cytokines were measured according to the manufacturer’s instructions by ELISA (Pierce Biotechnology Inc. Rockford, IL).

The murine cell line EL4, extensively used for studies with these Th TFs [20], [26], [27], [43] was obtained from Dr. N.W. Zwirner (Department of Microbiology, Parasitology and Immunology, School of Medicine, University of Buenos Aires, Buenos Aires, Argentina) and were treated, where indicated, with 10–100 nM Dex (a synthetic GC) or 0.1–10 µM CpdA and 10 ng/ml PMA, 500 ng/ml I, and/or 0.3 mM cAMP (all, except CpdA, from Sigma Chemical Co., St. Louis, MO). Transfection experiments were also repeated in human Jurkat T cells and the A20 mouse B lymphoma cell line, both obtained from Dr. Mirta Giordano (Department of Immunology, Institute for Hematologic Research, National Academy of Medicine, Buenos Aires, Argentina).

CpdA was synthesized as described by Louw et al. [44]. CpdA was lyophilized and stored at −70°C.

Some experiments were performed in the presence of the specific GR antagonist RU38486 (Sigma Chemical Co., St. Louis, MO) (1 µM), added 30 minutes before the addition of GCs, to prove ligand-specific interactions with the ligand-binding domain of GR.

For analysis of MAPK activation, cells were incubated with or without the PKA inhibitor H89, the p38 inhibitor SB203580, the MEK-1 inhibitor PD98059 and c-Jun NH2-terminal kinase (JNK) inhibitor SP600125 (10, 20 and 40 µM) (Calbiochem, San Diego, CA) for 1 h before stimulating with cAMP.

### Plasmids and transfection assays

The plasmid constructs were kindly provided and previously described as follows: the murine IL-5 promoter (which contains the –1200 to +33 sequence from the IL-5 gene) coupled to the luciferase reporter vector, was provided by Dr. T. Yokota [45] (IL-5-Luc); the murine wild-type IL-5 promoter (−120 to +44) and mutated on the GATA-3 binding site, were provided by Dr. E. Serfling [46] (IL-5-Luc-WT and IL-5-Luc-Gm); the murine GATA-3 expression vector, obtained from Dr. J. Leiden [11] (pcDNA3-GATA-3); the reporter gene vector carrying GATA-3 response elements, coupled to the luciferase reporter vector, was provided by Dr. A. Ray [18] (GATA-RE-Luc); the human GR expression vector subcloned in a cytomegalovirus promoter (CMV)-hGR and the CMV-β-galactosidase were supplied by Dr. D. Spengler [47]; the p38 MAPK expression vector (pcEFL-p38), the chimera pG5-Luc/ATF-2 and the catalytic subunit of PKAc was provided by Dr. O. Cosso and Dr. T. Tanos [48], [49]; the heat stable inhibitor of the PKA (PKI) was provided by Dr. R. Mauer [50]; the murine IFN-γ promoter was provided by Dr. H. S. Fox [51] and was subcloned into the pGL3-Basic luciferase reporter vector (Promega, Madison, WI) [26] (IFN-γ-Luc); murine pJG4.5mT-bet, obtained from Dr. L. H. Glimcher [21], was subcloned into the pcDNA3 expression vector (Invitrogen, Carlsbad, CA) [26] (pcDNA3-T-bet); the T-bet binding sites [21] subcloned into the pTATA-GL3-Basic luciferase reporter vector (Promega, Madison, WI) was obtained as previously described [26] ((T-bet-RE)3-Luc); the TK-GRE2-Luc promoter, was supplied by Dr. D. Spengler [47]; the wild-type human GR (phGR-SB) and its GR DNA-binding domain (DBD)-derived mutants A458T and S425G, were provided by Dr. A. C.B. Cato [30], the construct containing the multimerized NF-κB-binding sites linked to a minimal promoter upstream of the luciferase gene (κB-Luc promoter) was previously described [52].

Transfection of EL4, Jurkat and A20 cells (5 × 10^7^ cells/ml) was performed by electroporation, as previously described [26], [27]. Cells were washed with PBS and extracts were prepared with reporter lysis buffer (Promega, Madison, WI). After treatments, cells were harvested and luciferase activity was measured as previously described [26], [27], using the Luciferase measure kit (Promega, Madison, WI) with a Junior luminometer (Berlthod, Bad Wildbad, Germany). CMVhGR expression vector was cotransfected in EL4 cells in order to make them responsive to GCs. In all cases, cells were cotransfected with the RSV-β-galactosidase plasmid expression vector, used as control for transfection efficiency to standardize the results. Lysates from the transfections were also analyzed by Western Blot as described below.

### Western Blot Assays

Following the appropriate inductions, cells were washed once with PBS (pH 7.0), and lysates were prepared as previously described [26], [27]. The membranes were incubated with the mouse monoclonal anti-GATA-3 antibody (200 ng/ml) (Santa Cruz Biotechnology, Santa Cruz, CA), anti-phospho-GATA-3 antibody (Abcam Inc, Cambridge, UK) (dilution: 1∶500), followed by incubation with HRP-conjugated specific secondary antibodies (dilution: 1∶3000) (Bio-Rad Laboratories, Hercules, CA), and detection was performed with the ECL kit according to the manufacturer’s instruction (Pierce Biotechnology, Rockford, IL). The anti-GAPDH antibody (dilution: 1∶10000) (Abcam Inc, Cambridge, UK) was routinely used as a loading control. Anti-histone H3 antibody (dilution: 1∶20000) (Millipore, Billerica, MA) was used as a control for the efficiency of the nuclear fractionation and the anti-GAPDH antibody (dilution: 1∶10000) (Abcam Inc, Cambridge, UK) as a cytoplasmic fractionation control. To separate nuclei from cytoplasm, we proceeded as previously described [27]. To separate nuclei from cytoplasm, 1 × 10^7^ cells were washed in ice-cold phosphate-buffered saline and incubated for 10 min on ice in 200 µl of buffer A containing 10 mM HEPES, pH 7.9, 1.5 mM MgCl_2_, 10 mM KCl, 1 mM dithiothreitol, 2 mM orthovanadate, 0.5 mM phenylmethylsulfonyl fluoride and protease inhibitor cocktail (Roche Diagnostics, Mannheim, Germany). At the end of the incubation, a 0.1 volume of 1% Nonidet P-40 was added and lysates were centrifuged at 3000 xg for 5 min. Supernatants were collected and used as cytoplasmic extract. Pelleted nuclei were resuspended in 50 µl of lysis buffer containing 20 mM HEPES, pH 7.9, 25% v/v glycerol, 420 mM NaCl, 1.5 mM MgCl_2_, 0.2 mM EDTA, 1 mM dithiothreitol, 2 mM orthovanadate, 0.5 mM phenylmethylsulfonyl fluoride and protease inhibitor cocktail (Roche Diagnostics, Mannheim, Germany). After incubation for 30 min at 4°C with vigorous shaking, nuclei were centrifuged at 12000 xg for 10 min and the supernatants were collected and used as nuclear extract.

To analyze MAPK activation, blots were incubated with rabbit anti-phospho-p38 (p-p38) (Thr180/Tyr182) and rabbit anti-pan-p38 antibodies (dilution: 1∶1000) **(**Cell Signaling, Beverly, MA).

### Statistics

Statistics were performed by ANOVA in combination with the Scheffé ´s test. Data are shown as mean ± SEM.
