# Correction: Compound A, a Dissociated Glucocorticoid Receptor Modulator, Inhibits T-bet (Th1) and Induces GATA-3 (Th2) Activity in Immune Cells

**DOI:** 10.1371/annotation/12a8fc89-5f47-4bad-8863-863d99a0e52d

**Published:** 2012-05-23

**Authors:** Ana C. Liberman, Maria Antunica-Noguerol, Viviane Ferraz-de-Paula, Joao Palermo-Neto, Carla N. Castro, Jimena Druker, Florian Holsboer, Marcelo J. Perone, Sarah Gerlo, Karolien De Bosscher, Guy Haegeman, Eduardo Arzt

The affiliations for Eduardo Arzt are incomplete in the online version of the article. Eduardo Arzt's complete affiliations are:

1 Laboratorio de Fisiología y Biología Molecular, Departamento de Fisiología y Biología Molecular y Celular, Facultad de Ciencias Exactas y Naturales, Universidad de Buenos Aires and Instituto de Investigación Biomedicina de Buenos Aires (IBioBA)-CONICET-Partner Institute of the Max Planck Society, Buenos Aires, Argentina, and 3 Max Planck Institute of Psychiatry, Munich, Germany. The affiliations in the PDF are correct. 

